# A Morning in the Charite Hospital

**Published:** 1875-03-15

**Authors:** 


### A MORNING IN THE CHARITE HOSPITAL.

VIRCHOW.—Eight A.M., on a cold, winter's morning in Berlin, is *schrecklich* early, but if you wish to attend Prof. Virchow's lecture, you must find your way by that time to the Pathological Institute, which is built in one of the many quadrangles of the great Charity Hospital. On the lower floor are the museum and dissecting rooms; above, the room for microscopy, a reading room, in which is placed Prof. Virchow's immense library, to which his students have free access; and adjoining this, a lecture-room, elegant in hard woods and fresco. About the room, something in the fashion of a Grecian border, rectangulates a long table, with a miniature track running its whole length, not as a toy railroad, as it might seem, but as a means of transit for the score of microscopes which stand waiting at the farther end. Close by, are as many more bright red platters, heaped high with broken bits of poor humanity; for, as all who die in the great Charite must pass through this institute, there is never lack of material. To-day's platters, for instance, show carcinoma of the stomach, liver and œsophagus, pleuro-pneumonia, hydrocele, aortic sclerosis, carnification of the lungs, granular pharyngitis, renal cicatrices, and diphtheritic membranes, *ad salicitatem*, for just now diphtheria happens to be epidemic.

"Meine Herren," says a little, jaundiced, shabby object, that might easily be mistaken for an old clo' man; and without further introduction, Herr Prof. Rudolph Virchow takes up the first platter and begins a dissertation upon cancer; illustrating his theories by diagrams in colored chalks upon the blackboard and by appropriate specimens. At the same time, microscopic preparations to illustrate the histology of the subject under discussion, were traveling along the railway, of which we have just spoken. So pass two or three hours, according to the amount of material on hand, Virchow speaking continuously all the while, spicing his remarks every now and then with a growl at a dilatory student, or a fling at Christianity, to enjoy which he would go further out of his way than John Randolph is said to have done to kick a sheep.

FRERICHS.—*F. Th.*, 11–12, *Medical Clinic*, *Charite*, says the *stundenplan*, so hurry across the quadrangle, or you may be obliged to claim your seat *vi et armis*, if you can; for in spite of cards and numbered places, the best seats on the front rows are an almost daily cause of "unpleasantness" between America and Germany. To-day, victory sides with our glorious bird, and we post ourselves at the head of the camp bedstead, upon which has just been brought in, a woman with a head as big as a peck measure. Moon-faced, fatherly Frerichs removes the cotton batting, and we behold a most gorgeous case of facial erysipelas, with bullæ attached. Over this he lingers, as if loth to let so beautiful a specimen depart, telling us of erysipelas variegata, marginata, dermatosa, bullosa, etc., etc.; but at last directs that a cathartic should be given, and sends her away, having repacked her in cotton wool.

Next comes an unfortunate man, who has been suffering for nearly five weeks from intestinal war and subsequent blockade. Nearly the whole German pharmacopœia has been poured into him, and still the port will not open. Why? "Purulent exudation, paralysis of the *darmmusculature*, obstruction from gall stones, scybala, intussusception, incarceration, angulation and ulceration," are all suggested as possible causes, but which is to blame, will be shown certainly by the post-mortem. [Here to enliven the patient, drawings from similar cases, and sections from diseased intestines were passed about.] "In the meantime, gentlemen, we will try to diminish peristaltic action by small doses of opium. Will use large injections of tepid water, also; and if tympanitis becomes too great, will draw off the accumulated gases by means of an explorative needle."

*N. B.*—Two days later, patient furnished very interesting *obduction*.

